# Crystal structure of 2-isopropyl-4-meth­oxy-5-methyl­phenyl 4-methyl­benzene­sulfonate

**DOI:** 10.1107/S2056989018003110

**Published:** 2018-02-28

**Authors:** Yassine Laamari, Aziz Auhmani, My Youssef Ait Itto, Jean-Claude Daran, Abdelwahed Auhmani, Mostafa Kouili

**Affiliations:** a Laboratoire de Physico-Chimie Moléculaire et Synthèse Organique, Département de Chimie, Faculté des Sciences, Semlalia BP 2390, Marrakech 40001, Morocco; b Laboratoire de Chimie de Coordination, CNRS UPR8241, 205 route de Narbonne, 31077 Toulouse Cedex 04, France; c Laboratoire de Chimie Organique et Analytique, Faculté des Sciences et, Techniques, Université Sultan Moulay Slimane, BP 523, 23000 Béni-Mellal, Morocco

**Keywords:** crystal structure, organic synthesis, tosyl­ation of alcohols, drug synthesis

## Abstract

The title compound, C_18_H_22_O_4_S, an hemisynthetic product, was obtained by the tosyl­ation reaction of the naturally occurring meroterpene *p*-meth­oxy­thymol. The mol­ecule comprises a tetra­substitued phenyl ring linked to a toluene­sulfonate through one of its O atoms. In the crystal, C—H⋯O and C—H⋯π inter­actions link the mol­ecules, forming a three-dimensional network.

## Abstract 2

The title compound, an hemisynthetic product, was obtained by the tosyl­ation reaction of the naturally occurring meroterpene *p*-meth­oxy­thymol 1.

## Chemical context   

Tosyl­ation of alcohols is an important transformation in organic synthesis. This transformation is usually achieved with *p*-toluene sulfonyl chloride, which is very reactive (Greene & Wuts, 1999 ▸; Yoshida et *al.*, 1999 ▸). Tosyl­ate is an important functional group in organic synthesis as it makes a good leaving group (Wagner & Zokk, 1955 ▸; Sandler & Karo, 1983 ▸). Indeed, tosyl­ates are used as inter­mediates in the synthesis of several drugs (Kim *et al.*, 1995 ▸; Morgan *et al.*, 1997 ▸). Furthermore, they have also been found to possess important biological activities (Kacem *et al.*, 2002 ▸; Kaleemullah et *al.*, 2012 ▸).
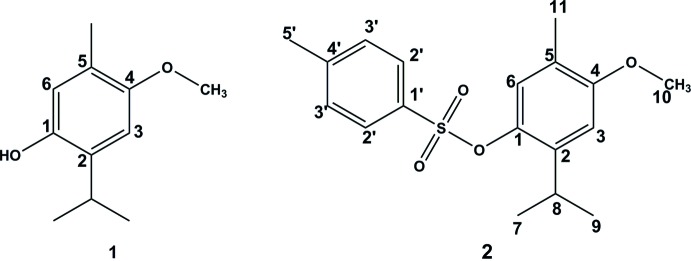


The hemisynthesis of 2-isopropyl-4-meth­oxy-5-methyl­phenyl 4-methyl­benzene­sulfonate **2** from naturally occurring *p*-meth­oxy­thymol **1** was undertaken with the aim of preparing meroterpenic tosyl­ate. X-ray single-crystal structure analysis allowed its full structure to be confirmed unambiguously.

## Structural commentary   

**Figure 1:**
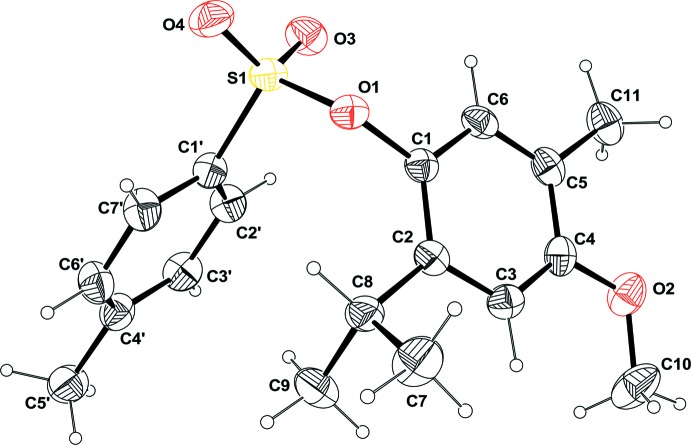
Mol­ecular view of compound **2** with the atom-labelling scheme. Displacement ellipsoids are drawn at the 50% probability level.

Compound **2** is built up from a tetra­substituted phenyl ring linked to a toluene­sulfonate unit through one of its oxygen atoms (Fig. 1 ▸). The two phenyl rings form a dihedral angle of 60.03 (9)°. Atoms S1 and C5′ are coplanar with the C1′–C6′ phenyl ring, their distances from the plane being 0.057 (3) and 0.031 (3) Å, respectively. Considering the connected atoms of the four substituents on the C1–C6 phenyl ring, three of them O2, C8 and C11 are roughly in the plane of the phenyl ring, deviating by only 0.011 (3), 0.014 (3) and 0.012 (3) Å, respectively, from the mean plane, whereas atom O1 is displaced slightly out of the plane by 0.101 (3) Å. This slight distortion might be related to the occurrence of a weak C—H⋯π inter­action between the C9 atom and the centroid *Cg*2 of the C1′–C6′ phenyl ring (Table 1 ▸).

## Supra­molecular features   

**Figure 2:**
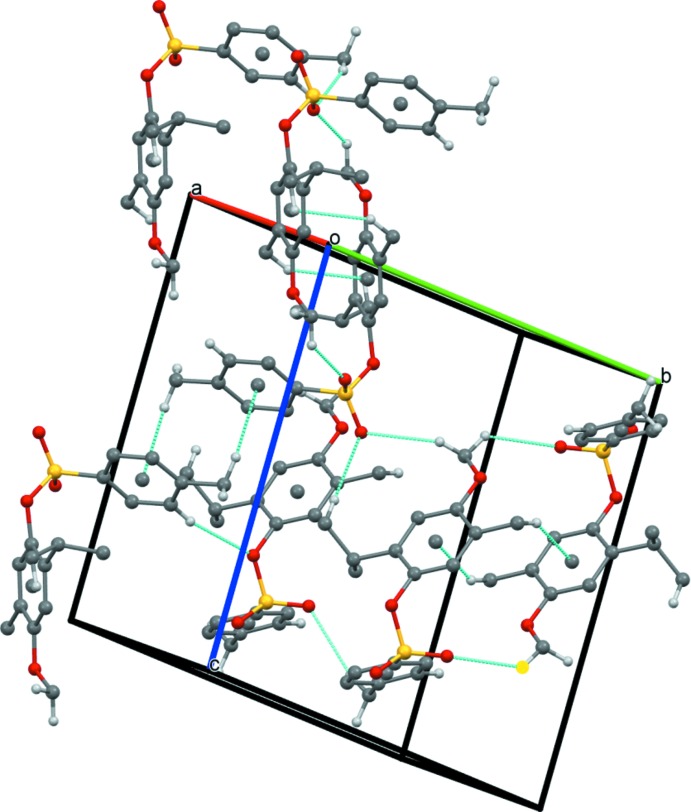
Partial packing view showing the C—H⋯O and C—H⋯π inter­actions (dotted lines). Only H atoms involved in hydrogen bonding are shown.

In the crystal, pairs of mol­ecules are linked though C—H⋯O inter­actions (Table 1 ▸), forming pseudo-dimer arranged around inversion centers (Fig. 2 ▸). Further C—H⋯O hydrogen bonds and C—H⋯π inter­actions (Table 1 ▸, Fig. 2 ▸) lead to the formation of a three-dimensional network.

## Database survey   

A search of the Cambridge Structural Database (CSD, version 5.38, last update May 2017; Groom *et al.*, 2016 ▸) for a tosyl­ate fragment bearing an organic substituent on one of its oxygen atoms revealed only three hits. Two of these compounds are closely related to compound **2**. The first, 5-bromo-2,3-di­methyl­phenol-1-(4-methyl­phenyl­sulfon­yloxy)benzene (KAWDAN; Niestroj *et al.*, 1998 ▸), is built up from a tosyl­ate attached to a phenyl ring substituted by two methyl groups and one bromine atom whereas the second, tetra­methyl-*p*-phenyl­ene *p*-di­toluene­sulfonate (TMPDTS; Wieczorek *et al.*, 1975 ▸), is built up from a tetra­methyl-substituted phenyl ring attached to two tosyl­ate units. A comparison of selected distances in compound **2** with those of two structures reveals that the geometries are very similar for all three compounds (Table 2 ▸). The most marked difference is the dihedral angle between the phenyl rings, 60.03 (9)° in **2** and 15.32 and 43.02° in KAWDAN and TMPDTS, respectively. The large dihedral angle in TMPDTS might be related to the occurrence of two bulky substituents on the central phenyl ring.

## Synthesis and crystallization   

In a 100mL flask, 430 mg (2.33mmol) of *p*-meth­oxy­thymol **1** were dissolved in 15 mL of pyridine and then 908 mg (4.66 mmol) of *para*-toluene­sulfonyl chloride were added. The reaction mixture was heated to reflux for two h. The end of the reaction was controlled by TLC. The reaction mixture was washed with a hydro­chloric acid solution (0.1 *M*) to neutral pH, extracted three times with ethyl ether (3 × 20 mL), dried over anhydrous Na_2_SO_4_ and concentrated under reduced pressure. The crude product was purified by silica gel column chromatography using hexa­ne/ethyl acetate (94:6) as eluent to give 360 mg (1.07 mmol, 46% yield) of 2-isopropyl-4-meth­oxy-5-methyl­phenyl 4-methyl­benzene­sulfonate **2**. X-ray quality colourless crystals were obtained by slow evaporation of a petroleum ether solution of the title compound.

**NMR data for compound 2:** ^1^H NMR (300 MHz, CDCl_3_): 6.66 (*s*, H-6), 6.84 (*s*, H-3), 3.45 (*sept*, H-8), 1.09 (*d*, H-9,H-10), 2.13 (*s*, H-7), 3.72 (*s*, H-10), 7.33 (*d*, H-3′), 7.75 (*d*, H-2′), 2.43 (s, H-5′) ppm. ^13^C NMR (75 MHz, CDCl_3_): 156.4 (C-1), 124.0 (C-2), 107.5 (C-3), 145.2 (C-4), 139.8 (C-5), 124.1 (C-6),15.7 (C-7), 26.8 (C-8), 23.1 (C-9, C-10), 55.4 (OCH_3_), 145.2 (C-1′), 129.7 (C-2′), 128.4 (C-3′), 139.7 (C-4′), 21.5 (C-5′) ppm.

## Refinement   

Crystal data, data collection and structure refinement details are summarized in Table 3 ▸. All H atoms were fixed geometrically and treated as riding with C—H = 1.0 (methine), 0.98 (meth­yl) or 0.95 Å (aromatic) with *U*_iso_(H) = 1.2*U*_eq_(CH and CH_2_) or *U*_iso_(H) = 1.5*U*_eq_(CH_3_).

## Supplementary Material

Crystal structure: contains datablock(s) I, global. DOI: 10.1107/S2056989018003110/xu5919sup1.cif

Supporting information file. DOI: 10.1107/S2056989018003110/xu5919Isup3.cml

Structure factors: contains datablock(s) I. DOI: 10.1107/S2056989018003110/xu5919Isup3.hkl

CCDC reference: 1825267

Additional supporting information:  crystallographic information; 3D view; checkCIF report

## Figures and Tables

**Table 1:** Hydrogen-bond geometry (Å, °) *Cg*1 and *Cg*2 are the centroids of the phenyl rings C1–C6 and C1′–C6′, respectively.

*D*—H⋯*A*	*D*—H	H⋯*A*	*D*⋯*A*	*D*—H⋯*A*
C3′—H3′⋯O1^i^	0.95	2.67	3.610 (2)	169
C5′—H5′3⋯O3^ii^	0.98	2.66	3.589 (3)	159
C7—H7*A*⋯O4^iii^	0.98	2.72	3.693 (3)	175
C10—H10*B*⋯O3^iv^	0.98	2.61	3.317 (3)	130
C10—H10*A*⋯O4^iii^	0.98	2.70	3.521 (3)	142
C5′—H5′2⋯*Cg*2^v^	0.98	2.84	3.706	148
C11—H11*B*⋯*Cg*1^iv^	0.98	2.75	3.635	150
C9—H9*A*⋯*Cg*2	0.98	2.70	3.5373	144

Symmetry codes: (i) 
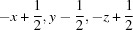
; (ii) 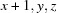; (iii) 
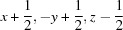
; (iv) 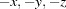; (v) 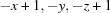.

**Table 2:** Selected structural parameters of compound **2** compared with closely related structures

	**2**	KAWDAN^*a*^	TMPDTS^*b*^
C1′—S1	1.749 (2)	1.748	1.732
S1—O1	1.597 (1)	1.598	1.599
O1—C1	1.428 (2)	1.425	1.428
C1′—S1—O1	104.76 (8)	98.83	102.37
S1—O1—C1	120.71 (11)	116.07	119.84
Dihedral angle	60.03 (9)	15.32	43.02

References: *a* Niestroj *et al.* (1998 ▸); *b* Wieczorek *et al.* (1975 ▸).

**Table 3:** Experimental details

Crystal data	
Chemical formula	C_18_H_22_O_4_S
*M*_r_	334.41
Crystal system, space group	Monoclinic, *P*2_1_/*n*
Temperature (K)	173
*a*, *b*, *c* (Å)	8.2226 (6), 14.5382 (9), 14.7230 (8)
β (°)	100.020 (6)
*V* (Å^3^)	1733.17 (19)
*Z*	4
Radiation type	Mo *K*α
μ (mm^−1^)	0.20
Crystal size (mm)	0.35 × 0.25 × 0.10

Data collection	
Diffractometer	Rigaku Oxford Diffraction Xcalibur Eos Gemini ultra
Absorption correction	Multi-scan (*CrysAlis PRO*; Rigaku OD, 2015 ▸)
*T*_min_, *T*_max_	0.791, 1.000
No. of measured, independent and observed [*I* > 2σ(*I*)] reflections	18172, 3532, 2777
*R*_int_	0.044
(sin θ/λ)_max_ (Å^−1^)	0.625

Refinement	
*R*[*F* ^2^ > 2σ(*F* ^2^)], *wR*(*F* ^2^), *S*	0.042, 0.110, 1.06
No. of reflections	3532
No. of parameters	213
H-atom treatment	H-atom parameters constrained
Δρ_max_, Δρ_min_ (e Å^−3^)	0.34, −0.34

Computer programs: *CrysAlis PRO* (Rigaku OD, 2015 ▸), *SIR97* (Altomare *et al.*, 1999 ▸), *SHELXL2014* (Sheldrick, 2015 ▸), *ORTEP-3 for Windows* (Farrugia, 1997 ▸) and *Mercury* (Macrae *et al.*, 2008 ▸).

## supplementary crystallographic information

### Crystal data

**Table:**

C_18_H_22_O_4_S	*F*(000) = 712
*M**_r_* = 334.41	*D*_x_ = 1.282 Mg m^−^^3^
Monoclinic, *P*2_1_/*n*	Mo *K*α radiation, λ = 0.71073 Å
*a* = 8.2226 (6) Å	Cell parameters from 4433 reflections
*b* = 14.5382 (9) Å	θ = 3.8–28.5°
*c* = 14.7230 (8) Å	µ = 0.20 mm^−^^1^
β = 100.020 (6)°	*T* = 173 K
*V* = 1733.17 (19) Å^3^	Box, colourless
*Z* = 4	0.35 × 0.25 × 0.10 mm

### Data collection

**Table:**

Rigaku Oxford Diffraction Xcalibur Eos Gemini ultra diffractometer	3532 independent reflections
Radiation source: Enhance (Mo) X-ray Source	2777 reflections with *I* > 2σ(*I*)
Graphite monochromator	*R*_int_ = 0.044
Detector resolution: 16.1978 pixels mm^-1^	θ_max_ = 26.4°, θ_min_ = 3.1°
ω scans	*h* = −10→10
Absorption correction: multi-scan (CrysAlis PRO; Rigaku OD, 2015)	*k* = −18→16
*T*_min_ = 0.791, *T*_max_ = 1.000	*l* = −18→18
18172 measured reflections	

### Refinement

**Table:**

Refinement on *F*^2^	0 restraints
Least-squares matrix: full	Hydrogen site location: inferred from neighbouring sites
*R*[*F*^2^ > 2σ(*F*^2^)] = 0.042	H-atom parameters constrained
*wR*(*F*^2^) = 0.110	*w* = 1/[σ^2^(*F*_o_^2^) + (0.0459*P*)^2^ + 0.814*P*] where *P* = (*F*_o_^2^ + 2*F*_c_^2^)/3
*S* = 1.06	(Δ/σ)_max_ < 0.001
3532 reflections	Δρ_max_ = 0.34 e Å^−^^3^
213 parameters	Δρ_min_ = −0.34 e Å^−^^3^

### Special details

**Table:**

Geometry. All esds (except the esd in the dihedral angle between two l.s. planes) are estimated using the full covariance matrix. The cell esds are taken into account individually in the estimation of esds in distances, angles and torsion angles; correlations between esds in cell parameters are only used when they are defined by crystal symmetry. An approximate (isotropic) treatment of cell esds is used for estimating esds involving l.s. planes.
Refinement. All H atoms attached to C atoms were fixed geometrically and treated as riding with C—H = 0.95 Å (aromatic), 0.98 Å (methyl), 1.0Å (methine) with U_iso_(H) = 1.2U_eq_(CH and C=CH) or U_iso_(H) = 1.5U_eq_(CH_3_).

### Fractional atomic coordinates and isotropic or equivalent isotropic displacement parameters (Å^2^)

**Table:**

	*x*	*y*	*z*	*U*_iso_*/*U*_eq_	
C1	0.0729 (2)	0.19454 (12)	0.12387 (12)	0.0244 (4)	
C1'	0.2295 (2)	0.11565 (13)	0.32646 (12)	0.0273 (4)	
C2'	0.2423 (2)	0.02563 (13)	0.29895 (13)	0.0319 (4)	
H2'	0.150817	−0.004394	0.262620	0.038*	
C2	0.2184 (2)	0.20290 (12)	0.08959 (12)	0.0253 (4)	
C3'	0.3907 (3)	−0.02031 (14)	0.32514 (13)	0.0351 (5)	
H3'	0.400221	−0.082269	0.306238	0.042*	
C3	0.2171 (2)	0.16786 (13)	0.00089 (12)	0.0281 (4)	
H3	0.314049	0.172671	−0.025740	0.034*	
C4	0.0786 (2)	0.12650 (13)	−0.04881 (12)	0.0283 (4)	
C4'	0.5259 (2)	0.02225 (14)	0.37844 (12)	0.0323 (4)	
C5	−0.0681 (2)	0.11891 (13)	−0.01288 (13)	0.0293 (4)	
C5'	0.6862 (3)	−0.02793 (16)	0.40828 (15)	0.0414 (5)	
H5'1	0.674385	−0.091812	0.386905	0.062*	
H5'2	0.714962	−0.026864	0.475724	0.062*	
H5'3	0.773618	0.002185	0.381667	0.062*	
C6	−0.0682 (2)	0.15452 (13)	0.07405 (13)	0.0284 (4)	
H6	−0.166024	0.151593	0.100070	0.034*	
C6'	0.5098 (2)	0.11365 (15)	0.40381 (13)	0.0356 (5)	
H6'	0.601735	0.144317	0.439007	0.043*	
C7	0.4269 (3)	0.32911 (16)	0.09104 (15)	0.0451 (6)	
H7A	0.459446	0.308452	0.033416	0.068*	
H7B	0.520807	0.359505	0.129571	0.068*	
H7C	0.334786	0.372516	0.077095	0.068*	
C7'	0.3633 (2)	0.16037 (14)	0.37888 (13)	0.0325 (4)	
H7'	0.353664	0.222483	0.397271	0.039*	
C8	0.3736 (2)	0.24634 (14)	0.14252 (13)	0.0310 (4)	
H8	0.349050	0.268405	0.203020	0.037*	
C9	0.5123 (3)	0.17540 (18)	0.16200 (16)	0.0483 (6)	
H9A	0.474804	0.122497	0.194085	0.072*	
H9B	0.608770	0.203173	0.200759	0.072*	
H9C	0.542309	0.155001	0.103658	0.072*	
C10	0.2226 (3)	0.08648 (18)	−0.17038 (15)	0.0526 (7)	
H10A	0.261778	0.149089	−0.178548	0.079*	
H10B	0.203927	0.054579	−0.229920	0.079*	
H10C	0.305633	0.053107	−0.126849	0.079*	
C11	−0.2193 (3)	0.07531 (15)	−0.06830 (14)	0.0388 (5)	
H11A	−0.307373	0.073020	−0.031237	0.058*	
H11B	−0.192880	0.012726	−0.085726	0.058*	
H11C	−0.256290	0.111763	−0.124059	0.058*	
O1	0.06333 (15)	0.23611 (8)	0.21065 (8)	0.0269 (3)	
O2	0.07192 (18)	0.09037 (10)	−0.13523 (9)	0.0391 (4)	
O3	−0.08697 (16)	0.10974 (10)	0.27106 (10)	0.0378 (3)	
O4	0.02806 (17)	0.24132 (10)	0.36634 (9)	0.0386 (4)	
S1	0.04212 (6)	0.17449 (3)	0.29760 (3)	0.02896 (14)	

### Atomic displacement parameters (Å^2^)

**Table:**

	*U*^11^	*U*^22^	*U*^33^	*U*^12^	*U*^13^	*U*^23^
C1	0.0260 (9)	0.0203 (9)	0.0269 (9)	0.0030 (7)	0.0041 (7)	0.0025 (7)
C1'	0.0316 (9)	0.0231 (10)	0.0270 (9)	0.0023 (8)	0.0049 (7)	0.0001 (7)
C2'	0.0354 (10)	0.0247 (10)	0.0349 (10)	−0.0030 (8)	0.0038 (8)	−0.0036 (8)
C2	0.0248 (9)	0.0208 (9)	0.0298 (9)	−0.0004 (7)	0.0029 (7)	0.0022 (7)
C3'	0.0445 (12)	0.0226 (10)	0.0392 (11)	0.0032 (9)	0.0098 (9)	−0.0032 (8)
C3	0.0297 (9)	0.0242 (10)	0.0313 (9)	−0.0007 (8)	0.0084 (7)	0.0022 (8)
C4	0.0363 (10)	0.0210 (10)	0.0262 (9)	0.0000 (8)	0.0016 (7)	0.0025 (7)
C4'	0.0379 (11)	0.0306 (11)	0.0287 (9)	0.0052 (9)	0.0067 (8)	0.0033 (8)
C5	0.0301 (9)	0.0206 (10)	0.0349 (10)	−0.0003 (8)	−0.0013 (8)	0.0063 (8)
C5'	0.0415 (12)	0.0441 (13)	0.0398 (11)	0.0126 (10)	0.0103 (9)	0.0081 (10)
C6	0.0230 (9)	0.0255 (10)	0.0366 (10)	0.0030 (8)	0.0045 (7)	0.0067 (8)
C6'	0.0355 (11)	0.0355 (12)	0.0331 (10)	0.0002 (9)	−0.0019 (8)	−0.0039 (9)
C7	0.0464 (13)	0.0481 (14)	0.0418 (12)	−0.0235 (11)	0.0107 (10)	−0.0079 (10)
C7'	0.0395 (11)	0.0236 (10)	0.0327 (10)	0.0021 (8)	0.0017 (8)	−0.0047 (8)
C8	0.0251 (9)	0.0385 (12)	0.0297 (9)	−0.0050 (8)	0.0057 (7)	−0.0050 (8)
C9	0.0276 (10)	0.0672 (17)	0.0477 (13)	0.0069 (11)	0.0003 (9)	−0.0045 (12)
C10	0.0641 (16)	0.0617 (16)	0.0368 (12)	−0.0238 (13)	0.0219 (11)	−0.0168 (11)
C11	0.0359 (11)	0.0347 (12)	0.0416 (11)	−0.0050 (9)	−0.0046 (9)	0.0020 (9)
O1	0.0272 (6)	0.0225 (7)	0.0322 (7)	0.0011 (5)	0.0083 (5)	0.0012 (5)
O2	0.0473 (9)	0.0394 (9)	0.0305 (7)	−0.0112 (7)	0.0062 (6)	−0.0057 (6)
O3	0.0309 (7)	0.0380 (8)	0.0450 (8)	−0.0052 (6)	0.0079 (6)	0.0068 (6)
O4	0.0411 (8)	0.0403 (8)	0.0376 (8)	0.0094 (7)	0.0153 (6)	−0.0038 (6)
S1	0.0277 (2)	0.0285 (3)	0.0323 (3)	0.0020 (2)	0.00963 (18)	0.00179 (19)

### Geometric parameters (Å, º)

**Table:**

C1—C2	1.382 (2)	C6—H6	0.9500
C1—C6	1.388 (2)	C6'—C7'	1.376 (3)
C1—O1	1.428 (2)	C6'—H6'	0.9500
C1'—C2'	1.379 (3)	C7—C8	1.526 (3)
C1'—C7'	1.390 (3)	C7—H7A	0.9800
C1'—S1	1.7486 (19)	C7—H7B	0.9800
C2'—C3'	1.386 (3)	C7—H7C	0.9800
C2'—H2'	0.9500	C7'—H7'	0.9500
C2—C3	1.400 (2)	C8—C9	1.527 (3)
C2—C8	1.513 (2)	C8—H8	1.0000
C3'—C4'	1.389 (3)	C9—H9A	0.9800
C3'—H3'	0.9500	C9—H9B	0.9800
C3—C4	1.380 (3)	C9—H9C	0.9800
C3—H3	0.9500	C10—O2	1.424 (3)
C4—O2	1.369 (2)	C10—H10A	0.9800
C4—C5	1.403 (3)	C10—H10B	0.9800
C4'—C6'	1.393 (3)	C10—H10C	0.9800
C4'—C5'	1.504 (3)	C11—H11A	0.9800
C5—C6	1.381 (3)	C11—H11B	0.9800
C5—C11	1.503 (3)	C11—H11C	0.9800
C5'—H5'1	0.9800	O1—S1	1.5967 (12)
C5'—H5'2	0.9800	O3—S1	1.4216 (14)
C5'—H5'3	0.9800	O4—S1	1.4221 (14)
			
C2—C1—C6	122.74 (16)	C8—C7—H7B	109.5
C2—C1—O1	118.38 (15)	H7A—C7—H7B	109.5
C6—C1—O1	118.64 (15)	C8—C7—H7C	109.5
C2'—C1'—C7'	120.86 (18)	H7A—C7—H7C	109.5
C2'—C1'—S1	120.13 (15)	H7B—C7—H7C	109.5
C7'—C1'—S1	119.00 (14)	C6'—C7'—C1'	119.26 (18)
C1'—C2'—C3'	118.97 (18)	C6'—C7'—H7'	120.4
C1'—C2'—H2'	120.5	C1'—C7'—H7'	120.4
C3'—C2'—H2'	120.5	C2—C8—C7	111.27 (16)
C1—C2—C3	116.24 (16)	C2—C8—C9	110.51 (17)
C1—C2—C8	123.71 (16)	C7—C8—C9	110.87 (17)
C3—C2—C8	120.05 (16)	C2—C8—H8	108.0
C2'—C3'—C4'	121.44 (18)	C7—C8—H8	108.0
C2'—C3'—H3'	119.3	C9—C8—H8	108.0
C4'—C3'—H3'	119.3	C8—C9—H9A	109.5
C4—C3—C2	121.63 (17)	C8—C9—H9B	109.5
C4—C3—H3	119.2	H9A—C9—H9B	109.5
C2—C3—H3	119.2	C8—C9—H9C	109.5
O2—C4—C3	123.76 (17)	H9A—C9—H9C	109.5
O2—C4—C5	114.99 (16)	H9B—C9—H9C	109.5
C3—C4—C5	121.26 (17)	O2—C10—H10A	109.5
C3'—C4'—C6'	118.20 (18)	O2—C10—H10B	109.5
C3'—C4'—C5'	121.55 (18)	H10A—C10—H10B	109.5
C6'—C4'—C5'	120.25 (18)	O2—C10—H10C	109.5
C6—C5—C4	117.33 (16)	H10A—C10—H10C	109.5
C6—C5—C11	121.84 (18)	H10B—C10—H10C	109.5
C4—C5—C11	120.83 (18)	C5—C11—H11A	109.5
C4'—C5'—H5'1	109.5	C5—C11—H11B	109.5
C4'—C5'—H5'2	109.5	H11A—C11—H11B	109.5
H5'1—C5'—H5'2	109.5	C5—C11—H11C	109.5
C4'—C5'—H5'3	109.5	H11A—C11—H11C	109.5
H5'1—C5'—H5'3	109.5	H11B—C11—H11C	109.5
H5'2—C5'—H5'3	109.5	C1—O1—S1	120.71 (11)
C5—C6—C1	120.79 (17)	C4—O2—C10	117.16 (15)
C5—C6—H6	119.6	O3—S1—O4	119.91 (9)
C1—C6—H6	119.6	O3—S1—O1	109.37 (8)
C7'—C6'—C4'	121.25 (18)	O4—S1—O1	102.77 (8)
C7'—C6'—H6'	119.4	O3—S1—C1'	109.08 (9)
C4'—C6'—H6'	119.4	O4—S1—C1'	109.79 (9)
C8—C7—H7A	109.5	O1—S1—C1'	104.76 (8)

### Hydrogen-bond geometry (Å, º)

Cg1 and Cg2 are the centroids of the phenyl rings C1–C6 and C1'–C6', respectively.

**Table:**

*D*—H···*A*	*D*—H	H···*A*	*D*···*A*	*D*—H···*A*
C3′—H3′···O1^i^	0.95	2.67	3.610 (2)	169
C5′—H5′3···O3^ii^	0.98	2.66	3.589 (3)	159
C7—H7*A*···O4^iii^	0.98	2.72	3.693 (3)	175
C10—H10*B*···O3^iv^	0.98	2.61	3.317 (3)	130
C10—H10*A*···O4^iii^	0.98	2.70	3.521 (3)	142
C5′—H5′2···*Cg*2^v^	0.98	2.84	3.706	148
C11—H11*B*···*Cg*1^iv^	0.98	2.75	3.635	150
C9—H9*A*···*Cg*2	0.98	2.70	3.5373	144

Symmetry codes: (i) −*x*+1/2, *y*−1/2, −*z*+1/2; (ii) *x*+1, *y*, *z*; (iii) *x*+1/2, −*y*+1/2, *z*−1/2; (iv) −*x*, −*y*, −*z*; (v) −*x*+1, −*y*, −*z*+1.
